# SCFAs switch stem cell fate through HDAC inhibition to improve barrier integrity in 3D intestinal organoids from patients with obesity

**DOI:** 10.1016/j.isci.2023.108517

**Published:** 2023-11-23

**Authors:** Mona Farhadipour, Kaline Arnauts, Mathias Clarysse, Theo Thijs, Kathrin Liszt, Bart Van der Schueren, Laurens J. Ceulemans, Ellen Deleus, Matthias Lannoo, Marc Ferrante, Inge Depoortere

**Affiliations:** 1 Gut Peptide Research Lab, Translational Research for Gastrointestinal Disorders (TARGID), KU Leuven, 3000 Leuven, Belgium; 2 Inflammatory Bowel Disease, Translational Research for Gastrointestinal Disorders (TARGID), KU Leuven, 3000 Leuven, Belgium; 3 Leuven Intestinal Failure and Transplantation (LIFT) Center, University Hospitals Leuven, 3000 Leuven, Belgium; 4 Department of Endocrinology, University Hospitals Leuven, 3000 Leuven, Belgium; 5 Department of Abdominal Surgery, University Hospitals Leuven, 3000 Leuven, Belgium; 6 Department of Gastroenterology and Hepatology, University Hospitals Leuven, 3000 Leuven, Belgium

**Keywords:** Biological sciences, Neuroscience, Behavioral neuroscience

## Abstract

Stem cells are a keystone of intestinal homeostasis, but their function could be shifted during energy imbalance or by crosstalk with microbial metabolites in the stem cell niche. This study reports the effect of obesity and microbiota-derived short-chain fatty acids (SCFAs) on intestinal stem cell (ISC) fate in human crypt-derived intestinal organoids (enteroids). ISC fate decision was impaired in obesity, resulting in smaller enteroids with less outward protruding crypts. Our key finding is that SCFAs switch ISC commitment to the absorptive enterocytes, resulting in reduced intestinal permeability in obese enteroids. Mechanistically, SCFAs act as HDAC inhibitors in stem cells to enhance Notch signaling, resulting in transcriptional activation of the Notch target gene HES1 to promote enterocyte differentiation. In summary, targeted reprogramming of ISC fate, using HDAC inhibitors, may represent a potential, robust therapeutic strategy to improve gut integrity in obesity.

## Highlights

• Obesity promotes proliferation of crypt based cells and impairs their differentiation
• SCFAs act as HDAC inhibitors to activate Notch signaling affecting stem cell fate
• Lineage commitment to absorptive enterocytes improves gut integrity in obesity
• Targeting stem cell biology is a beneficial strategy to reshape the gut epithelium

## Graphical abstract

**Figure:**
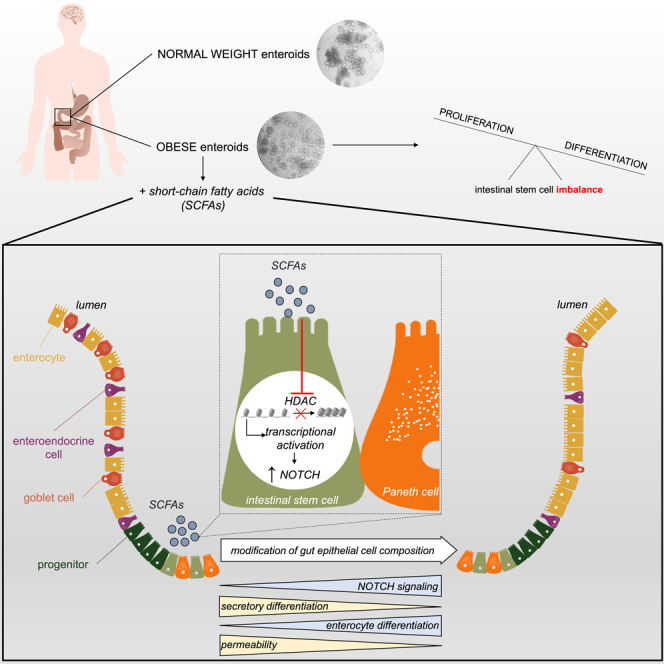

## Introduction

Gut homeostasis relies on the proper functioning of the intestinal barrier. This barrier consists of the outer mucus layer with the commensal gut microbiota, chemical substances (digestive secretions, antimicrobial peptides, and cytokines), the central epithelial cell layer with specialized epithelial cells and the inner lamina propria where innate and adaptive immune cells reside. The interplay between these different layers is essential to maintain a balanced permeability.^1^ The epithelial lining is composed of absorptive cells, primarily enterocytes lined by tight junctions responsible for nutrient absorption and gut integrity, and secretory cells such as enteroendocrine cells (EECs) releasing appetite regulating hormones, goblet cells producing mucins, and Paneth cells releasing antimicrobial peptides and stem cell niche-derived signals.^2^

These intestinal epithelial cells, with the exception of Paneth cells, maintain an unusually high turnover rate of 3–4 days. This rapid self-renewal process is maintained by intestinal stem cells (ISCs) that reside at the base of intestinal crypts intermingled between Paneth cells. These actively cycling, LGR5+ crypt-based cells can self-renew and/or give rise to a population of highly proliferative transit-amplifying cells that will differentiate into specific cell lineages. Stem cell fate determination is regulated by a balanced interplay between the Wnt/β-catenin signaling and Notch signaling, with transcription factor ATOH1 promoting secretory differentiation and HES1 repressing ATOH1 to drive absorptive lineage commitment.^3^^,^^4^ The crypt-region also contains quiescent ISCs that can transform into actively cycling ISCs upon tissue damage. Growth factors secreted from Paneth cells or subepithelial myofibroblasts near ISCs are part of the stem cell niche and activate signaling pathways that control the balance between stem cell self-renewal and differentiation.^5^

Several studies have shown that also the nutritional status is a major determinant of stem cell behavior.^6^ Caloric restriction promotes the preservation and self-renewal of ISCs after sensing of nutritional deprivation by mTORC1 in mice.^7^ A more recent study attributed the enhanced stemness after short-term fasting to the activation of peroxisome proliferator-activated receptor (PPAR) family targets, more specifically PPAR-δ, which is a major inducer of fatty acid β-oxidation (FAO).^8^ Interestingly, a high-fat diet (HFD) in mice promoted tumorigenesis by enhancing ISC number, proliferation and function through the same pathway involving PPAR-α/δ induced FAO.^9^^,^^10^ Recent studies showed that an obesogenic diet not only induced ISC hyperproliferation, but also caused a dysregulation in the network of transcription factors controlling epithelial cell differentiation and thus the composition of the mature epithelial cell types in mice and humans.^11^^,^^12^

Obesity is not only an established risk factor for cancer but is also characterized by disturbed gut hormone release, which affects energy homeostasis.^13^ Moreover, a leaky gut due to an increased gut permeability promotes bacterial translocation and systemic inflammation in these patients.^14^ Cani et al. was the first to describe a positive correlation between gut dysbiosis and increased gut permeability.^15^ Interestingly, individuals with obesity are characterized by reduced levels of butyrate-producing bacteria, which are essential for upregulating tight junction proteins and hence preserving intestinal barrier function.^14^^,^^16^ Indeed, studies in HFD-induced obese mice showed that supplementation with oligofructose, which is fermented by gut microbiota to short-chain fatty acids (SCFAs) such as butyrate, decreased gut permeability and body weight gain.^17^

Moreover, SCFAs also act as modulators of ISC homeostasis. Several studies found that SCFAs affected the proliferation of LGR5+ stem cells and modulated lineage commitment to the secretory cells.^18^^,^^19^^,^^20^^,^^21^ Specifically, oligofructose was shown to increase the number of GLP-1 containing EECs (L-cells) by upregulation of enteroendocrine progenitors in the colon of rats.^22^ This finding was later confirmed in mouse and human enteroids, where exposure to SCFAs increased the number of L-cells.^23^

Taken together, these findings indicate that stem cell biology is influenced not only by the energy status of the host (e.g., obesity), but also by microbial metabolites (e.g., SCFAs). However, most studies have been conducted in mice and the mechanism by which SCFAs affect human ISC homeostasis has not been studied. This work aims to fill this gap in our knowledge and may lead to mechanistic insights important for restoring dysregulated gut health in patients with obesity. To this end, primary crypt-derived 3D intestinal organoids (enteroids) from normal weight individuals and patients with obesity were developed as a platform to study human stem cell biology. This model recapitulates essential features of the *in vivo* tissue architecture. We aim to investigate whether 1) the phenotype and ISC lineage commitment of human enteroids is affected by obesity and 2) SCFAs affect ISC differentiation and hence epithelial composition and function (permeability). SCFAs exert their biological effects through the activation of free fatty acid receptors (FFAR2-FFAR3) and the inhibition of histone deacetylases (HDACs).^24^ The presence of FFAR2 on human ISCs and the effect of HDAC inhibition by ketones on ISC homeostasis in mice, prompted us to study whether SCFAs can modulate human ISC biology using similar mechanisms.^25^^,^^26^^,^^27^

## Results

To investigate the effect of obesity on stem cell homeostasis, enteroids from normal weight individuals and patients with obesity were characterized during the expansion phase (undifferentiated crypt-based cells) and differentiation phase (stem cell lineage commitment).

### Obesity affects the phenotype of enteroids during the expansion phase

**Figure 1:**
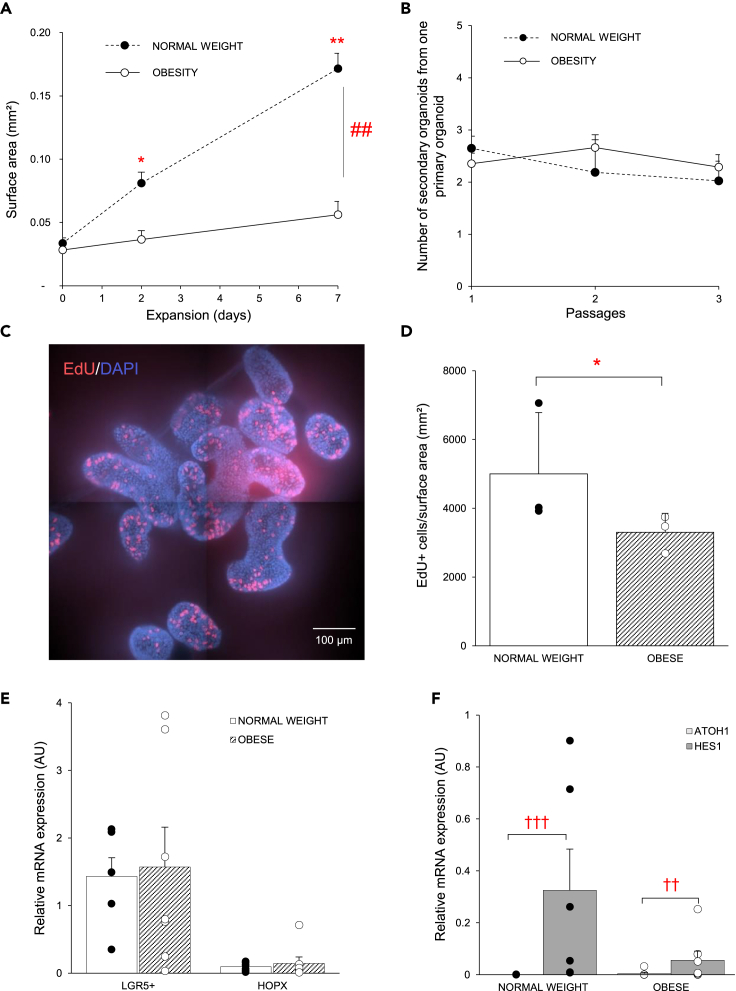
Obesity affects the phenotype of enteroids during the expansion phase (A) Time-dependent changes in surface area of enteroids from both study groups (n_normalweight_ = 3, n_obese_ = 4). (B) Clonogenicity of enteroids at day 2 of expansion from both enteroid populations (n_normalweight_ = 3, n_obese_ = 4). (C) Representative EdU staining (Alexa Fluor 647, red) in enteroids from a patient with obesity. Nuclei were stained with DAPI (blue). (D) Average number of EdU+ cells/surface area (mm^2^) in enteroids from both study groups (n_normalweight_ = 3, n_obese_ = 3). (E) The relative mRNA expression (2^−ΔΔCt^) of stem cell markers *LGR5+* and *HOPX* and (F) the relative mRNA expression (2^−ΔCt^) of the absorptive (*HES1*) and secretory (*ATOH1*) transcription factor in both enteroid populations (n_normalweight_ = 6, n_obese_ = 7). Data are presented as mean ± SEM. Statistical significance was determined using a mixed model with patient as the random effect for the surface area measurements and qPCR analysis, and a non-paired, two-tailed Student’s *t* test for the EdU proliferation assay. n is the number of individuals. ∗p < 0.05, ∗∗p < 0.05 versus obese, ##p < 0.01 time versus population, ††p < 0.01, †††p < 0.001 versus HES1.

During expansion, the surface area of enteroids increased in a time-dependent manner, but the size of enteroids from patients with obesity was significantly smaller (population x time: p < 0.01) than that of enteroids from normal weight individuals from day 2 onwards (Figures 1A, and S1A). No difference in organoid-forming capacity (clonogenicity) of ISCs was observed between both enteroid populations (Figure 1B). Cell proliferation assay with 5-ethynyl-2′-deoxyuridine (EdU) showed that enteroids from patients with obesity have a lower number of EdU^+^ cells/enteroid surface area (mm^2^) (p < 0.05) compared to the normal weight group at the end of the expansion phase (day 7) (Figures 1C, 1D; and S2A). This did not result in an effect of obesity on the mRNA expression levels of *LGR5+* (active) ISCs or *HOPX* (quiescent) ISCs (Figure 1E). No effect of obesity was found on the mRNA expression of *OLFM4* and *SOX9* positive crypt-based cells (Figure S3).

Even though stem cell renewal is favored during expansion, commitment to one of the two cell lineages already starts to take place. In enteroids from both populations, the mRNA expression of the transcription factor *HES1* that induces the program of absorptive cell differentiation was significantly higher (p < 0.05) than that of *ATOH1* that drives secretory cell differentiation (Figure 1F). In addition, *HES1* mRNA expression tended (p = 0.06) to be higher in enteroids from normal weight individuals. Our data indicate that stem cells in enteroids from both populations retain their predisposition toward the absorptive progenitors, whereas stem cells from normal-weight individuals show higher proliferation, which translates into a faster stem cell fate decision toward the absorptive cell lineage.

### Obesity affects the phenotype of enteroids during the differentiation phase

**Figure 2:**
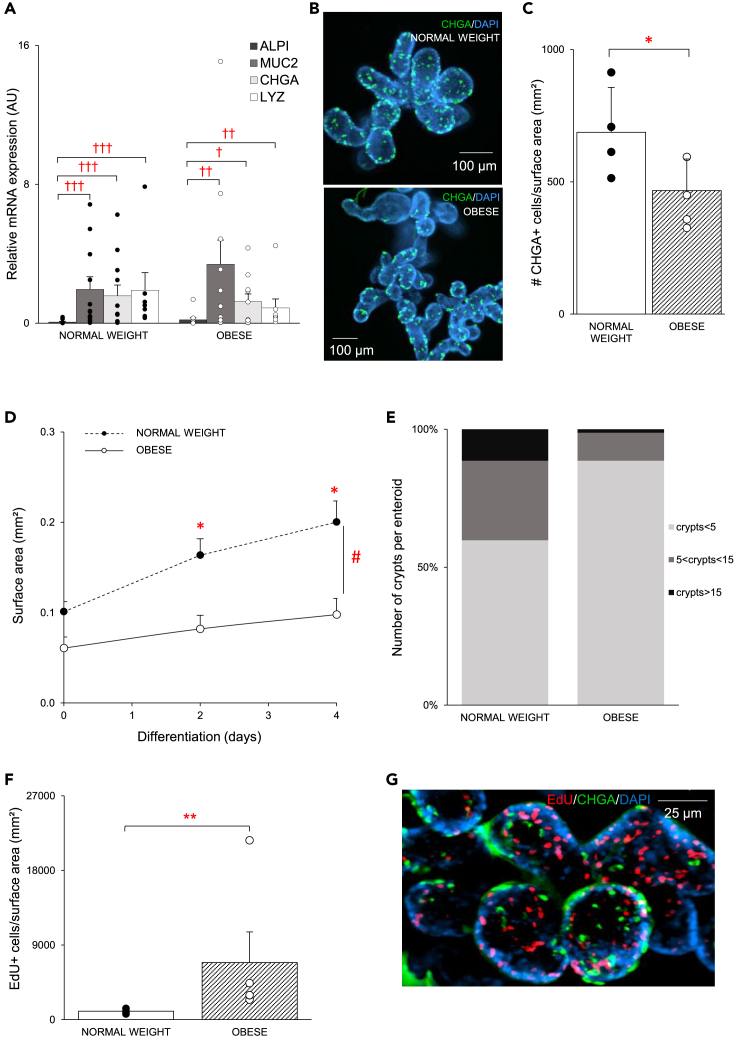
Obesity affects the phenotype of enteroids during the differentiation phase (A) The relative mRNA expression (2^−ΔCt^) of the absorptive cell marker (*ALPI*) and secretory cell markers (*MUC2, CHGA, LYZ*) in both study groups (n_normalweight_ = 7–11, n_obese_ = 8–11) at day 4 of differentiation. (B) Representative image of a whole-mount immunofluorescence staining of CHGA+ (CY3, green) cells in enteroids from a patient with obesity and a normal weight individual. Nuclei were stained with DAPI (blue). (C) The number of CHGA+ cells at day 4 of differentiation in enteroids from patients with obesity and normal weight individuals (n_normalweight_ = 4, n_obese_ = 4). (D) Time-dependent changes in surface area of enteroids from normal weight individuals and patients with obesity during differentiation (n_normalweight_ = 4, n_obese_ = 3). (E) Number of crypt formations per enteroid at day 4 of differentiation in enteroids from both populations (n_normalweight_ = 4, n_obese_ = 4). (F) Average number of EdU+ cells/surface area (mm^2^) in enteroids from normal weight individuals and patients with obesity (n_normalweight_ = 3, n_obese_ = 5). (G) Representative image of a whole-mount immunofluorescence staining of EdU (Alexa Fluor 647, red) and CHGA (CY3, green) in enteroids from a patient with obesity. Nuclei were stained with DAPI (blue). Data are presented as mean ± SEM. Statistical significance was determined using a mixed model with patient as the random effect for surface area measurements and qPCR analysis, and a non-paired, two-tailed Student’s *t* test for the EdU proliferation assay and for the quantification of the immunofluorescence staining. n is the number of individuals. †p < 0.05, ††p < 0.01, †††p < 0.001 versus ALPI. ∗p < 0.05 versus obese. #p < 0.05 time versus population.

To promote differentiation of enteroids to the secretory lineage, including EECs, Wnt3a was removed from the cell culture medium and the γ-secretase inhibitor, DAPT, was added during the differentiation phase. This decreased the mRNA expression of the absorptive enterocyte marker *ALPI* and increased the expression of the secretory EEC marker chromogranin A (*CHGA*) (p < 0.05), the goblet cell marker mucin 2 (*MUC2*) (p < 0.01) and the Paneth cell marker lysozyme (*LYZ*) (p < 0.01) in both enteroid populations (Figure 2A). Although obesity did not affect *CHGA* mRNA expression, a significant reduction (p < 0.05) in the population of CHGA+ cells was found at the end of differentiation in enteroids from patients with obesity compared to normal weight individuals (Figures 2B and 2C).

During differentiation, the surface area of enteroids from patients with obesity remained smaller (population x time: p < 0.05) compared to normal weight individuals (Figures 2D, and S1B). Moreover, crypt development, a measure of the differentiation process in organoids, differed between both populations.^28^ Less enteroids with more than five crypt formations were observed in enteroids from patients with obesity compared to normal weight individuals (Figure 2E). In addition, more EdU^+^ cells/surface area (mm^2^) (p < 0.05) were found at the end of differentiation in the obese enteroid population (Figures 2F, and S2B). These positive cells did not co-localize with differentiated CHGA^+^ cells, indicating that mature EECs do not undergo cell proliferation and that EdU^+^ cells are representing proliferating crypt-based cells (Figure 2G). Taken together, these data imply that stem cells from patients with obesity are less prone to differentiate than those from normal-weight individuals.

### The enteroendocrine cell signature from the primary tissue is preserved in enteroids during the differentiation phase

**Figure 3:**
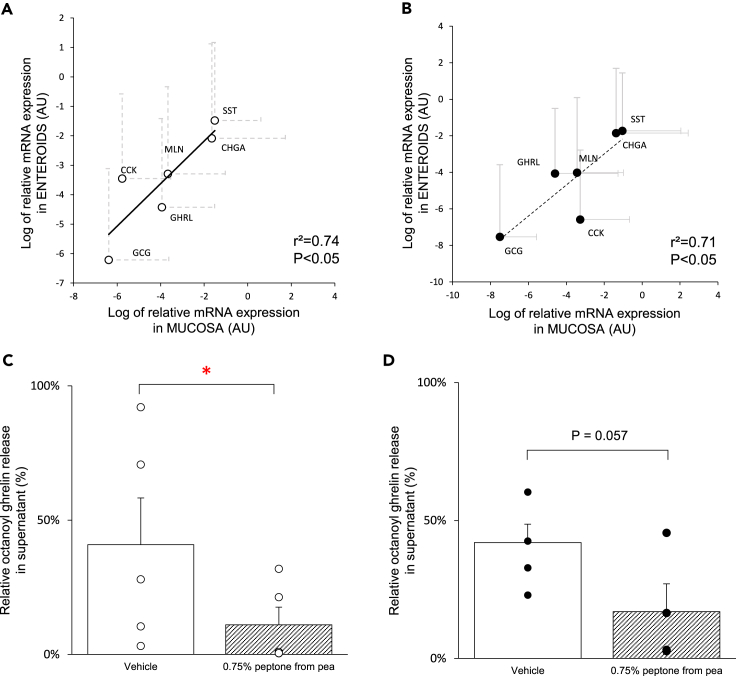
Enteroids mimic the EEC signature from the primary tissue during the differentiation phase (A and B) Correlation of the relative mRNA expression (2^−ΔCt^) of the different EEC markers between the mucosa and enteroids of (A) patients with obesity (n = 7) and (B) normal weight individuals (n = 7). Data are presented as mean ± SEM. (C and D) Effect of 3 h stimulation with 0.75% peptone from pea on octanoyl ghrelin release in the supernatant of enteroids (C) from patients with obesity (n = 5) and (D) normal weight individuals (n = 4). The octanoyl ghrelin release was expressed as a fraction of total hormone content per well. Data are presented as mean ± SEM. Statistical significance was determined by a Pearson correlation analysis for the expression of gut hormones in the mucosa versus enteroids, and a paired, two-tailed Student’s *t* test for ghrelin radioimmunoassay. n is the number of individuals. ∗p < 0.05 versus vehicle.

To assess if EEC features from the primary tissue were kept in the enteroid model, the mRNA expression levels of the gut hormones chromogranin A (*CHGA*), ghrelin (*GHRL*), motilin (*MLN*), cholecystokinin (*CCK*), glucagon-like peptide 1 (*GCG*) and somatostatin (*SST*) in the mucosa of each individual were correlated with their corresponding mRNA expression levels in the generated enteroids. A significant correlation was found between the expression of these genes in the mucosa and in the enteroids from patients with obesity (r^2^ = 0.74, p < 0.05) or normal weight individuals (r^2^ = 0.71, p < 0.05) (Figures 3A and 3B). These findings confirm that enteroids keep the EEC signature from the primary tissue.

To investigate if enteroids were functionally active, the release of octanoyl ghrelin was measured in response to 0.75% peptone, a pea hydrolysate that mimics the effects of a meal. Indeed, 0.75% peptone decreased (p < 0.05) octanoyl ghrelin release with 64 ± 20% in the supernatant of enteroids from patients with obesity, indicating that enteroids responded to the feeding stimulus (Figure 3C). Similar findings were observed in enteroids from normal weight individuals (Figure 3D).

**Figure 4:**
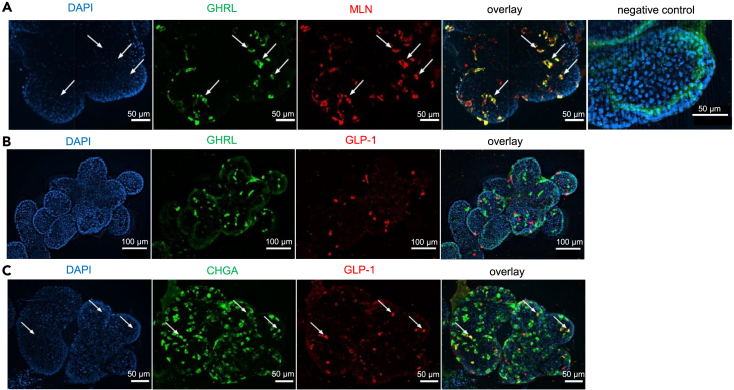
Representative whole-mount immunofluorescence staining for gut hormones in enteroids from a patient with obesity during the differentiation phase Representative double-immunofluorescence images of (A) GHRL+ cells (CY3, green) and MLN+ cells (Cy5, red), (B) GHRL+ cells (CY3, green) and GLP-1+ cells (Cy5, red), and (C) CHGA+ cells (CY3, green) and GLP-1+ cells (Cy5, red) in enteroids from a patient with obesity. Nuclei were labeled with DAPI (blue). Arrows indicate co-localization.

Representative whole-mount immunostaining of enteroids confirmed the (co)-expression of several EEC markers at the protein level (Figure 4). In enteroids from patients with obesity, 58 ± 12% of GHRL+ cells co-localized with MLN+ cells (Figure 4A), while no co-localization was found between GHRL+ and GLP-1+ cells (Figure 4B). This confirms previous findings that gut hormones with a similar function in appetite regulation are co-localized.^29^^,^^30^^,^^31^ In addition, 53 ± 12% of GLP-1+ cells co-localized with CHGA+ cells (Figure 4C).

### SCFAs switch stem cell differentiation toward the absorptive cell lineage at the expense of the secretory lineage

**Figure 5:**
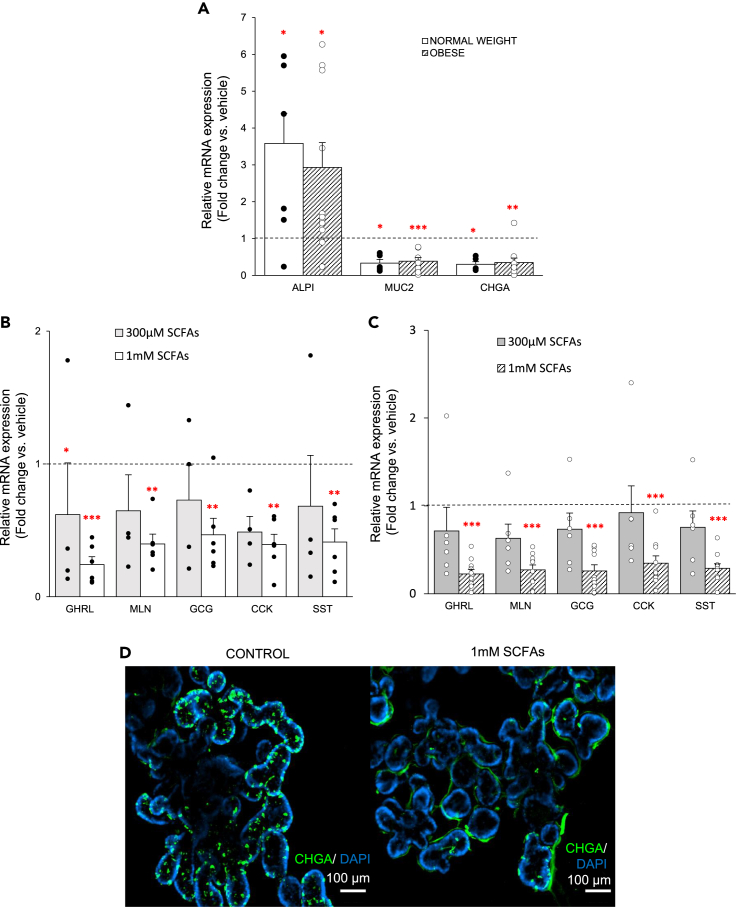
SCFAs switch stem cell fate toward the absorptive cell lineage in enteroids from normal weight individuals and patients with obesity during the differentiation phase (A–C) (A) Effect of SCFAs (1 mM) on the relative mRNA expression of absorptive enterocytes (*ALPI*) and secretory epithelial cells (*MUC2, CHGA*) during the differentiation phase in both enteroid populations (n_normalweight_ = 6, n_obese_ = 10). (B-C) Concentration-dependent effect of SCFAs on the relative mRNA expression of the gut hormones in (B) normal weight individuals (n_300μM_ = 4, n_1mM_ = 6) and (C) patients with obesity (n_300μM_ = 6, n_1mM_ = 10). (D) Representative image of a whole-mount immunofluorescence staining of CHGA+ (CY3, green) cells at the end of differentiation in enteroids from a patient with obesity after treatment with SCFAs (1 mM). Nuclei were labeled with DAPI (blue). Data are presented as mean ± SEM. Relative mRNA expression is presented as fold change vs. vehicle (2^−ΔΔCt^). Statistical significance was determined using a non-paired, two-tailed Student’s *t* test for qPCR analysis of general gut epithelial cell markers. For the effect on gut hormones a mixed model with patient as the random effect was used. n is the number of individuals. ∗p < 0.05, ∗∗p < 0.01, ∗∗∗p < 0.001 versus vehicle.

Crosstalk between microbial metabolites and ISCs was investigated by determining the effect of treatment with 1 mM SCFAs (3:1:1 ratio acetate, propionate and butyrate) on ISC commitment during the differentiation phase in enteroids from both study groups. In enteroids from patients with obesity, SCFAs (1 mM) increased (p < 0.05) *ALPI* mRNA expression 2.9-fold, but decreased (p < 0.01) *MUC2* (0.4-fold) and *CHGA* (0.3-fold) mRNA expression. (Figure 5A). The same effect was observed in enteroids from normal weight individuals.

The concentration-dependent effect of SCFAs on the gut hormone mRNA expression was investigated in more detail. SCFAs at 1 mM, but not at 300 μM, decreased the mRNA expression of the hunger hormones *GHRL* and *MLN,* the satiety hormones *GCG* and *CCK,* and the inhibitory hormone *SST* in enteroids from both study groups (Figures 5B and 5C). The effect of 1 mM SCFAs on stem cell fate did not differ between both populations. The magnitude of the SCFA-induced inhibition did not differ between the orexigenic genes (*GHRL, MLN*) and the anorexigenic genes (*GCG, CCK*) in both populations. Whole-mount immunostaining confirmed that protein expression for the pan-EEC marker, CHGA, was reduced (p < 0.05) with 80 ± 2% after treatment with 1 mM SCFAs at day 4 of differentiation in enteroids from patients with obesity (Figure 5D). SCFAs did not affect the morphological properties (size, crypt development) nor the number of proliferating EdU+ cells of enteroids from patients with obesity, thereby ensuring that SCFAs affect stem cell differentiation and not proliferation (Figures S4A–S4C).

### SCFAs act as HDAC inhibitors to activate the Notch signaling pathway and switch stem cell fate

**Figure 6:**
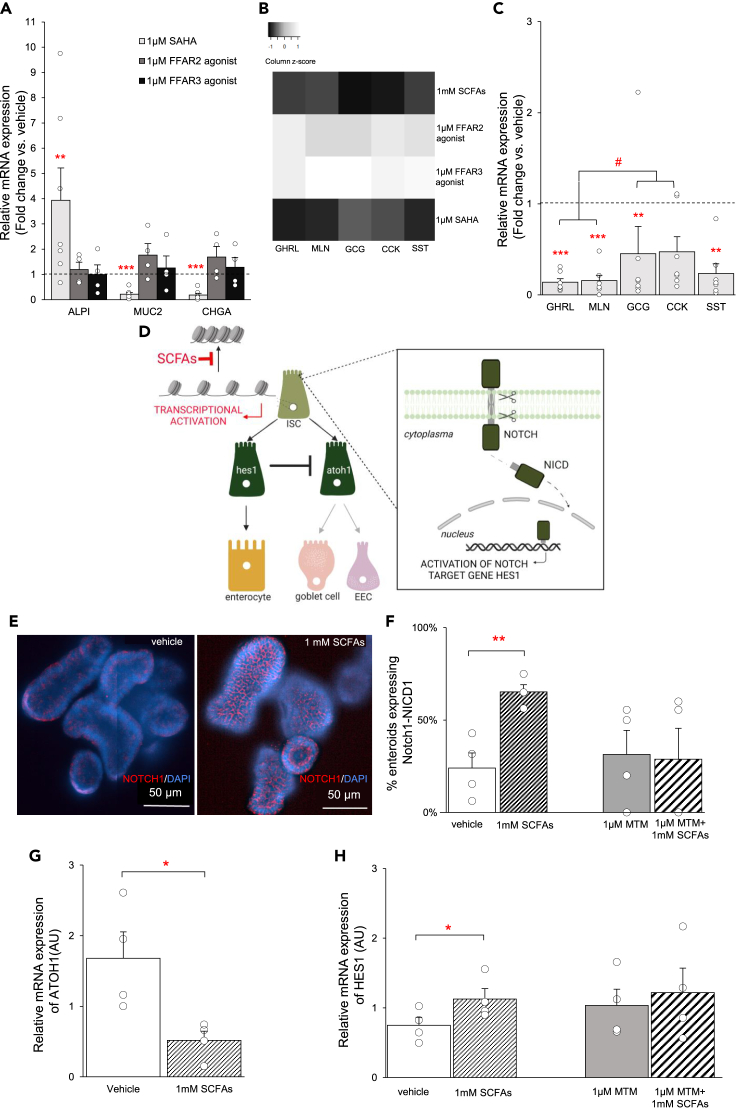
SCFAs act as HDAC inhibitors to activate Notch signaling and Notch target genes (A) Effect of 1 μM SAHA, 1 μM FFAR2 agonist and 1 μM FFAR3 agonist on the relative mRNA expression (fold change vs. vehicle, 2^−ΔΔCt^) of absorptive enterocytes (*ALPI*) and secretory epithelial cells (*MUC2, CHGA*) in enteroids from patients with obesity (n_SAHA_ = 7, n_FFAR2_ = 4, n_FFAR3_ = 4). (B) Heatmap representation of the effect of SCFAs compared to the effect of FFAR2 agonist, FFAR3 agonist and SAHA on the relative mRNA expression (fold change vs. vehicle, 2^−ΔΔCt^) of gut hormones in enteroids from patients with obesity (n_SCFAs_ = 10, n_FFAR2_ = 4, n_FFAR3_ = 4, n_SAHA_ = 7). Heatmap ranges from dark (fold change <1) to light (fold change >1). (C) Effect of 1 μM SAHA on the fold change in relative mRNA expression (fold change vs. vehicle, 2^−ΔΔCt^) of the gut hormones in enteroids from patients with obesity (n = 7). (D) Schematic overview of SCFAs acting as HDAC inhibitors to activate the Notch signaling pathway and the Notch target gene HES1 to switch lineage commitment to the absorptive enterocytes. (E) Representative image of a whole-mount immunofluorescence staining of the Notch1 intracellular domain (CY5, red) in enteroids from a patient with obesity stimulated with vehicle or SCFAs (1 mM). Nuclei were stained with DAPI (blue). (F) Effect of 1 mM SCFAs in the absence or presence of mithramycin A (MTM) on the number of enteroids from patients with obesity (n = 4) expressing Notch1-NICD1 after 32 h of differentiation. (G) Effect of 1 mM SCFAs on the relative mRNA expression (2^−ΔΔCt^) of *ATOH1* in the obese enteroid population (n_SCFAs_ = 4) after 32 h of differentiation. (H) Effect of 1 mM SCFAs in the absence or presence of 1 μM mithramycin A (MTM) on the relative mRNA expression (2^−ΔΔCt^) of *HES1* in the obese enteroid population ((n_SCFAs_ = 4, _nMTM+SCFAs_ = 4) after 32 h of differentiation. Data are presented as mean ± SEM. Statistical significance was determined using a mixed model with patient as the random effect for the effect of all stimulants on the general gut epithelial cell markers and for the effect of SAHA on gut hormones. For the quantification of the immunofluorescence staining and qPCR analysis of the transcription factors HES1 and ATOH1, a paired, two-tailed Student’s *t* test was used. n is the number of individuals. ∗p < 0.05, ∗∗p < 0.01, ∗∗∗p < 0.001 versus vehicle. #p < 0.05 versus anorexigenic genes.

Two major signaling mechanisms have been identified for SCFAs: activation of free fatty acid G protein-coupled receptors (FFARs) or inhibition of histone deacetylases (HDAC).^24^ To assess the contribution of these signaling pathways, differentiation was initiated in the presence of a FFAR2 agonist ((S)-2-(4-chlorophenyl)-3,3-dimethyl-N-(5-phenylthiazol-2-yl)butanamide, 1 μM), a FFAR3 agonist (AR420626, 1 μM) and a broad-spectrum class I/II/IV HDAC inhibitor, SAHA (1 μM). In enteroids from patients with obesity, treatment with the HDAC inhibitor SAHA induced an increase (p < 0.05) in *ALPI* (3.9-fold) mRNA expression and a decrease (p < 0.01) in *MUC2* (0.2-fold) and *CHGA* (0.2-fold) mRNA expression (Figure 6A). These markers were not affected upon treatment with FFAR2 or FFAR3 agonists. Similar to SCFAs, SAHA also downregulated the mRNA expression of the gut hormones, with the exception of CCK (Figures 6B and 6C). However, the suppressive effect of SAHA on the expression of orexigenic genes (*GHRL*, *MLN*) was more pronounced (p < 0.05) than this on the anorexigenic genes (*GCG*, *CCK*) (Figure 6C).

We propose that SCFAs function as HDAC inhibitors in stem cells, activating the Notch signaling pathway. This would subsequently lead to the activation of the absorptive transcription factor HES1 that acts as a negative regulator of the secretory transcription factor ATOH1 to increase enterocyte expression (Figure 6D).^27^^,^^32^ To evaluate this hypothesis, the effect of SCFAs was assessed after 32 h of differentiation instead of in fully differentiated enteroids (96 h). Whole-mount immunostaining for the intracellular domain of Notch1 (NICD1), which represents the active form of Notch1, revealed a 42% ± 6% increase (p < 0.05) in the number of enteroids expressing Notch1-NICD1 after treatment with 1 mM SCFAs (Figures 6E and 6F). However, SCFAs did not affect Notch signaling after pretreatment with 1 μM mithramycin A (MTM), an inhibitor of HDAC inhibition. These findings confirm that SCFAs act as HDAC inhibitors to activate the Notch signaling pathway. As a result, the mRNA expression of the secretory transcription factor *ATOH1* was decreased (p < 0.05) (Figure 6G), whereas the absorptive transcription factor *HES1* was increased (p < 0.05) after treatment with 1 mM SCFAs (Figure 4H). No effect on *HES1* mRNA expression was found after pretreatment with 1 μM MTM (Figure 6H).

### SCFAs improve gut integrity by reprogramming stem cell fate to absorptive enterocytes

**Figure 7:**
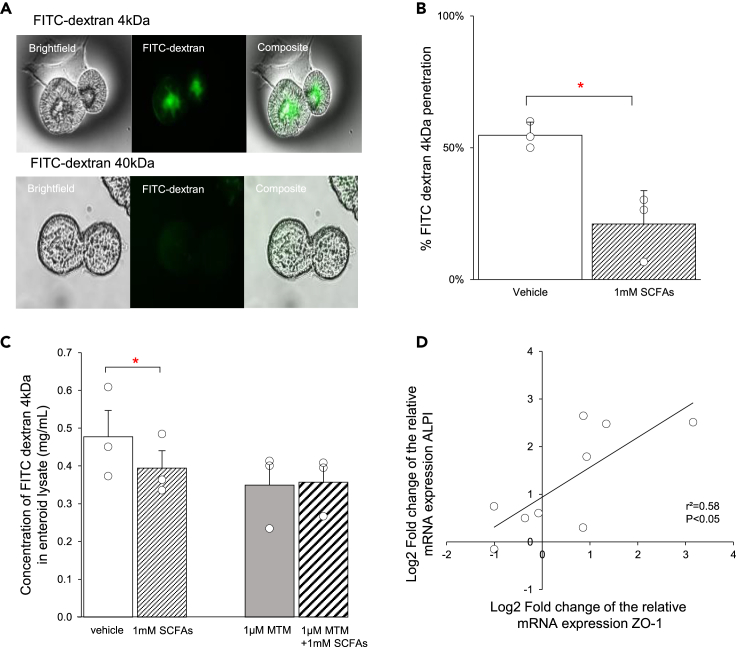
SCFAs improve gut barrier integrity by switching ISC differentiation to the absorptive enterocytes (A) Representative image of FITC dextran 4 kDa and 40 kDa diffusion in the lumen of enteroids from a patient with obesity. (B) Effect of 1 mM SCFAs on the permeability of FITC dextran (4 kDa) into the lumen of enteroids from patients with obesity (n = 3) after 4 days of differentiation. (C) Quantitative measurement of FITC dextran (4 kDa) in the enteroid lysate after stimulation with 1 mM SCFAs in the absence or presence of 1 μM mithramycin A (MTM) in enteroids from patients with obesity (n = 3) after 32 h of differentiation. (D) Pearson correlation analysis of the fold change in the relative mRNA expression (vs. vehicle, 2^−ΔΔCt^) between the absorptive enterocytes (*ALPI*) and the tight junction protein zonula occludens-1 (*ZO-1*) of enteroids from patients with obesity (n = 9) after treatment with 1 mM SCFAs after 4 days of differentiation. Data are presented as mean ± SEM. Statistical significance on permeability was determined with a paired, two-tailed Student’s *t* test n is the number of individuals. ∗p < 0.05 versus vehicle.

Next, we assessed if the SCFA-induced increase in the mRNA expression of enterocytes (*ALPI*)decreased the permeability of the epithelial layer in enteroids. The fluorescent tracer FITC dextran (4 kDa), but not FITC dextran (40 kDa), quickly (30 min) labeled the enteroid lumen indicating a high permeability of enteroids for small compounds (Figure 7A). In the presence of 1 mM SCFAs, the permeability of FITC dextran (4 kDa) into the lumen of enteroids from patients with obesity was reduced (p < 0.05) with 38 ± 13% in fully differentiated enterocytes (Figure 7B). Interestingly, after 32 h of differentiation, during which stem cell commitment was evaluated, the SCFAs-induced decrease (16 ± 2%) in gut permeability was not observed after pretreatment with 1 μM MTM (Figure 7C). These findings underscore the role of HDAC inhibition in maintaining gut integrity.

In addition, the SCFA-induced upregulation in the mRNA expression of *ALPI* correlated (r^2^ = 0.58, p < 0.05) with the upregulation of the tight junction protein zonula occludens-1 *(ZO-1)* in enteroids from patients with obesity (Figure 7D). These findings indicate that SCFAs enhance expression of enterocytes and their associated tight junctions to improve gut barrier integrity.

## Discussion

This study shows that the nutritional status of the host influences the dynamics of the human ISC niche. Obesity slows down the differentiation process of ISCs leading to smaller enteroids with less developed crypt formations, less CHGA positive EECs and more proliferative crypt-based cells. In addition, microbial metabolites in the microenvironment of stem cells reprogram stem cell fate. SCFAs switch stem cell commitment to the absorptive lineage resulting in decreased enteroid permeability. This is orchestrated by SCFA-induced HDAC inhibition of ISCs that activates the Notch signaling pathway. Notch-induced HES1 transcription promotes absorptive lineage commitment of stem cells. We conclude that HDAC inhibitors or pre-/probiotic interventions that increase SCFA levels may be beneficial to improve intestinal epithelial integrity in patients with obesity. Moreover, our findings illustrate that modulation of the number of gut epithelial cells is an interesting therapeutic approach that might result in a longer retention, requiring less frequent drug administration.

In contrast to studies in HFD induced obese mice, we could not confirm an increase in ISC renewal.^9^^,^^10^ Indeed, in enteroids from patients with obesity mRNA expression of stem cell markers was not affected and the number of proliferating crypt-based cells was decreased during expansion of stem cells. However, during differentiation the lineage commitment to mature enteroids was delayed in the obese enteroid population as evidenced by an increased population of non-mature, proliferating crypt-based cells. This proliferative state may predispose patients with obesity to develop cancer.^33^ The impaired differentiation process in enteroids from patients with obesity was also evidenced by less outward protruding crypts and resulted in an enteroid population with less CHGA+ cells. The latter confirms previous findings in HFD-induced mice and in intestinal biopsies from patients with obesity and may point to altered appetite regulation.^12^^,^^13^ Our data further confirm that enteroids recapitulate the EEC signature from the primary tissue in both normal weight individuals and patients with obesity. Although EECs were initially classified by a letter code, each linked to a single hormone, several reports including real-time single-cell differentiation mapping showed that EECs were bi- or tri-hormonal.^30^^,^^31^^,^^34^ Immunofluorescence studies showed that ghrelin and motilin but not GLP-1 are co-expressed by the same EEC population in the enteroid model, which confirms previous findings reported in tissue sections from human and porcine small intestine.^29^ The release of ghrelin was decreased after stimulation with pea peptone, used to mimic a meal, which is consistent with our previous results in a gastric ghrelinoma cell line and in human duodenal segments.^35^^,^^36^

In the second part of this study, we focused on the modulatory role of microbial metabolites on the stem cell niche during homeostasis and obesity. A randomized trial showed that dietary fibers that promote SCFA-producing strains alleviated type 2 diabetes and reduced body weight partly via increased production of GLP-1.^37^ Chambers et al. showed that acute delivery of an inulin-propionate ester to the human colon induced an acute release of PYY and GLP-1 and reduced energy intake.^38^ Interestingly, long-term delivery prevented body weight gain but effects on the release of satiety hormones were absent. Although the acute effect is probably mediated via direct activation of FFAR2 on L-cells, we hypothesized that SCFAs might also act via FFAR2 receptors expressed on stem cells to affect lineage commitment on the long term and change the number of EECs and hence their plasma level.^17^^,^^39^ Indeed, in our enteroid model SCFAs pushed stem cell commitment to the absorptive lineage, however, independent from FFAR2, and resulted in a downregulation of the expression of orexigenic and anorexigenic gut hormones. This effect was observed in both enteroid populations, suggesting that the effect of SCFAs is not affected by obesity. It is unlikely that this will influence appetite behavior or gastric emptying in patients with obesity since the SCFA-induced downregulation was not EEC selective and did therefore not affect the gut hormone balance.

Our data indicate that SCFAs function as HDAC inhibitors in stem cells to switch lineage commitment since the effect of SCFAs was mimicked by the HDAC inhibitor, SAHA. However, in contrast to SCFAs, SAHA was more selective in inhibiting differentiation toward the hunger hormones rather than to the satiety hormones. Both SCFA and SAHA act as a class I/II HDAC inhibitor but SAHA additionally inhibits class IV HDACs (HDAC11).^40^^,^^41^ A recent *in vivo* study in mice showed that selective inhibition of HDAC11 also promotes the development of brown adipose tissue and “browning” of white adipose tissue in obesity.^42^ In addition, the use of HDAC6 (class IIb) inhibitors in HFD induced obese mice led to weight loss by increasing central leptin sensitivity.^43^ These findings suggest a potential future application of targeted HDAC inhibition in weight management for individuals with obesity.

Contrary to our findings, Petersen et al. reported that SCFAs selectively upregulated transcription factors associated with the enteroendocrine lineage and enhanced differentiation toward L-cells in mouse and human small intestinal organoids.^23^ The higher concentration of SCFAs (7 mM at 3.5:0.75:0.75 ratio versus 1 mM at 3:1:1 ratio in our study) used and the early termination of the differentiation process (2 days versus 4 days in our study) in their study may account for these differences. Single-cell transcriptomic studies of the small intestine in mice and humans revealed differences in the kinetics of stem cell lineage commitment to EECs, and showed that all EEC sub lineages were only detectable after 70h of differentiation.^31^^,^^34^ These findings together with the fact that the gut epithelium renews itself every four to five days, suggest that a differentiation process of four days is considered as the ideal endpoint for readout.

Prebiotic fibers, such as inulin or oligofructose, are fermented to SCFAs and have protective effects on intestinal barrier dysfunction in patients with obesity but also in patients with inflammatory bowel disease.^37^^,^^44^ SCFAs have been described to reinforce the mucous layer through upregulation of mucin 2, thereby enhancing protection against luminal pathogens.^45^^,^^46^ In addition, studies have shown that SCFAs increase the expression of tight junction proteins and the reassembly of tight junctions through AMPK.^47^ The former hypothesis was not confirmed in our study since SCFAs downregulated the expression of MUC2 in our enteroid model. Our data support the latter hypothesis since SCFAs upregulated the expression of enterocytes, which correlated with the expression of the tight junction protein, zonula occludens-1. This resulted in a decreased entry of FITC dextran (4 kDa) in the lumen of human enteroids and thus a decreased permeability.

Our findings reveal for the first time the mechanism involved in the SCFA-induced shift in ISC lineage commitment. Previous findings demonstrated the interplay between HDAC and Notch signaling pathway, and highlighted the transcriptional repression of Notch target genes by HDAC activity.^27^^,^^48^ Our study confirmed that SCFAs ats as HDAC inhibitors in stem cells to trigger the activation of the Notch signaling pathway. This subsequently induced the expression of the Notch target gene HES1, thereby repressing the secretory transcription factor ATOH1. As a result, lineage decision was skewed to the absorptive enterocytes that decreased enteroid permeability.

In conclusion, enteroids from patients with obesity mimic the epithelial composition of the native tissue and can be used to study the therapeutic effect of metabolites or pharmacological drugs on stem cell biology that determines intestinal epithelial homeostasis and function. Our study suggests that increasing SCFA levels through pre- or probiotics, or use of HDAC inhibitors such as SAHA may not only be beneficial in improving intestinal barrier dysfunction, but may also lead to a more favorable gut hormone balance that may reduce appetite signaling in patients with obesity. Future *in vivo* studies should reveal whether a more tailored approach using different classes of HDAC inhibitors may be warranted to improve also metabolic dysfunctions beyond the leaky gut in patients with obesity.

### Limitations of the study

The human enteroid model is valuable because it consists of multiple cell types present in the corresponding epithelium and represents a more complex system than cell lines. Nevertheless, it lacks vascularization, microbial or viral interactions and cell to cell communication with stromal cells and immune cells. Therefore, several apical and basolateral signals affecting ISC homeostasis *in vivo* are not present. This may affect the efficacy of microbial metabolites (e.g., SCFA) to alter the epithelial composition and improve gut integrity. To address this, co-culture strategies should be included. In addition, the utilization of a CRISPR-Cas9-induced gene knockout enteroid model would enable a more comprehensive investigation into the transcription factors underlying the effect of SCFAs in modulating lineage commitment.

Furthermore, the absence of sex and gender-based analyses may affect the generalizability of our findings.

## STAR★Methods

### Key resources table

**Table:** 

REAGENT or RESOURCE	SOURCE	IDENTIFIER
**Antibodies**		

Goat anti-chromogranin A	Santa Cruz	Cat#SC-1488; RRID: AB_2276319
Goat anti-ghrelin	Santa Cruz	Cat#SC-10368; RRID: AB_2232479
Rabbit anti-GLP1	Abcam	Cat#Ab108443; RRID: AB_10866149
Rabbit anti-motilin	Inge Depoortere	N/A
Rabbit anti-Notch1	Cell Signaling Technology	Cat#D1E11; RRID: AB_2153354
Donkey anti-goat CY3	Jackson ImmunoResearch	Cat#715-165-150; RRID: AB_2340813
Donkey anti-rabbit CY5	Jackson ImmunoResearch	Cat#711-175-152; RRID: AB_2340607
DAPI	ThermoFisher Scientific	Cat#D1306; RRID: AB_2629482

**Chemicals, peptides, and recombinant proteins**		

Matrigel®	Corning	Cat#356231
Advanced DMEM-F12	ThermoFisher Scientific	Cat#12634010
Hepes	ThermoFisher Scientific	Cat#15630056
Glutamax	ThermoFisher Scientific	Cat#35050061
Penicillin-Streptomycin Solution	ThermoFisher Scientific	Cat#15140130
Wtn3a	Stem Cell Institute KULeuven	N/A
R-spondin	Stem Cell Institute KULeuven	N/A
Noggin	Stem Cell Institute KULeuven	N/A
EGF	ThermoFisher Scientific	Cat#PMG8041
A83-01	Tocris Bioscience	Cat#2939
SB202190	Sigma-Aldrich	Cat#S7067
Nicotinamide	Sigma-Aldrich	Cat#N0636
N-Acetyl-L-Cysteine	Sigma-Aldrich	Cat#A9165
B27	ThermoFisher Scientific	Cat#17504044
Y27632	Sigma-Aldrich	Cat#Y0503
Recovery™ freezing medium	ThermoFisher Scientific	Cat#12648010
CHIR99201	Sigma-Aldrich	Cat#SML1046
DAPT	Tocris BioScience	Cat#2634
Acetate	Sigma-Aldrich	Cat#S2889
Propionate	Sigma-Aldrich	Cat#P1880
Butyrate	Sigma-Aldrich	Cat#B5887
FFAR2-agonist	Sigma-Aldrich	Cat#371725
FFAR3-agonist AR420626	Sigma-Aldrich	Cat#SML1339
SAHA	Sigma-Aldrich	Cat#SML0061
Mithramycin A	ThermoFisher Scientific	Cat#10599213
Normal donkey serum	Jackson Immunoresearch	Cat#017-000-121
Triton X-100	Sigma-Aldrich	Cat#11332481001
Mowiol®-488	Sigma-Aldrich	Cat#83181
Peptone from Pea	Fluka Analytical	Cat#96174
EDTA-free protease inhibitor cocktail	ThermoFisher Scientific	Cat#87785
FITC-dextran 4 kDa	TdB Labs AB	Cat#FD4
FITC-dextran 40 kDa	TdB Labs AB	Cat#FD40
CitiFluor™	ThermoFisher Scientific	Cat#50-302-34

**Critical commercial assays**		

ReliaPrep miRNA Cell and Tissue Miniprep System	Promega	Cat#Z6212
RNAeasy minikit	Qiagen	Cat#74004
LightCycler® 480 SYBR Green I Master	Roche Diagnostics	Cat#04707516001
Click-it® EdU assay (647)	ThermoFisher Scientific	Cat#C10640

**Oligonucleotides**		

Primers (see Table S2)	Sigma-Aldrich	N/A

**Software and algorithms**		

ImageJ	N/A	https://imagej.nih.gov/ij/

**Other**		

PluriStrainer® 100μm	PluriSelect Life Science	Cat#SKU43-50100-51
96-CELLSTAR® plate	Greiner	Cat#655090

### Resource availability

#### Lead contact

Further information and requests for resources and reagents should be directed to and will be fulfilled by the lead contact, Inge Depoortere (Inge.Depoortere@kuleuven.be).

#### Materials availability

This study did not generate new unique reagents.

#### Data and code availability

• All data reported in the paper are available from the lead contact upon request.
• This paper does not report original code.
• Any additional information related to this study is available from the lead contact upon request.

### Experimental methods and study participant details

#### Tissue collection and study approval

Full-thickness jejunal tissue was obtained from normal weight multi-organ donors (age<65y, BMI<26 kg/m^2^, non-diabetic, donation after circulatory death [DCD] or brain death [DBD]) and from patients with obesity who underwent Roux-en Y gastric bypass surgery (age<65y, BMI>35kg/m2, non-diabetic). Human tissue was obtained in collaboration with the Leuven Intestinal and Failure Transplant LIFT and the bariatric surgery team of the University Hospitals Leuven. The study was approved by the Medical Ethics Committee UZ KU Leuven/Research (approval number S56978 [organ donors] and S57826 [patients with obesity]) and performed in accordance with the declaration of Helsinki. All patients with obesity gave written informed consent prior to study inclusion. Detailed protocols for human tissue procurement have been published previously.^35^ Patient and organ donor demographics are shown in Table S1.

#### Generation of enteroids

The mucosa was dissected from the muscle layer in ice-cold phosphate buffered saline (PBS), pH 7.4, and incubated with 2 mM EDTA in chelation buffer, pH 7.4, (5.6 mM Na_2_HPO_4_, 8.0 mM KH_2_PO_4_, 96.2 mM NaCl, 1.6 mM KCl, 43.4 mM sucrose, 54.9 mM D-sorbitol, 0.5 mM DL-dithiothreitol) for 40-45 min at 4°C.^49^ Jejunal crypts and villi were then scraped off and crypts were isolated after filtration using a 100 μm PluriStrainer® (PluriSelect Life Science) and seeded in 1:1 ratio Matrigel® (Corning) and ADF+++ (advanced DMEM-F12 with 1% HEPES, 1% glutamax, 1% of a 10,000 units penicillin and 10 mg streptomycin/mL solution) (ThermoFisher Scientific). Enteroids were generated after seven days in expansion medium (EM) consisting of 50% v/v Wnt3A (conditioned medium), 20% v/v R-spondin (conditioned medium), 10% v/v Noggin (conditioned medium), 50 ng/mL EGF (ThermoFisher Scientific), 500 nM A83-01 (Tocris Bioscience), 10 mM SB202190 (Sigma-Aldrich), 10 mM nicotinamide (Sigma-Aldrich), 1.25 mM n-acetylcysteine (Sigma-Aldrich), 1X B27 (ThermoFisher Scientific) in ADF+++. The conditioned medium was kindly provided by the Stem Cell Institute of KU Leuven. The Rho kinase-inhibitor Y27632 (10 μM, Sigma-Aldrich) was added during the first 2 days in culture. After 7 days, enteroids were collected in 6-10 cryotubes, depending upon the number of developed enteroids, with Recovery™ Freezing Medium (ThermoFisher Scientific) and stored in liquid nitrogen.

### Method details

#### Culturing of enteroids

Frozen enteroids were thawed and cultured in EM with the addition of Y27632 and the Wnt- activator CHIR99201 (1 μM, Sigma-Aldrich) for the first two days. Enteroids were passaged every 7 days and used for 4 passages. Differentiation was commenced after 4-5 days in EM by changing to differentiation medium (DM) (EM without 50% v/v Wnt3A, 10 mM SB202190 and 10 mM nicotinamide, but with the addition of 3 μM tert-Butyl (S)-(2S)-2-[2-(3,5-difluorophenyl)acetamido]propanamido-phenylacetate (DAPT, Tocris Bioscience)). During the first two days of differentiation, the enteroids were stimulated with one of the following components: 0.3 or 1 mM SCFAs at a 3:1:1 ratio^50^ for acetate, propionate and butyrate (Sigma-Aldrich), 1 μM FFAR2-agonist (S)-2-(4-chlorophenyl)-3,3-dimethyl-N-(5-phenylthiazol-2-yl)butanamide (Sigma-Aldrich), 1 μM FFAR3-agonist AR420626 (Sigma-Aldrich) or 1 μM suberoylanilide hydroxamic acid (SAHA) (Sigma-Aldrich). After two days, the medium was changed back to DM without stimulants for two more days, after which enteroids were harvested for analysis. A role for HDAC inhibition was studied by pretreatment of enteroids with 1 μM mithramycin A (MTM) (Thermofisher Scientific) or vehicle for 2 hours, followed by stimulation with SCFAs in the absence or presence of MTM for another 32 hours, after which enteroids were harvested for analysis.

#### Morphology

The morphology of enteroids was monitored during the expansion and differentiation phase (minimum 3 passages) in the absence or presence of stimulants. Pictures were taken from all enteroids within a fixed area in three wells per passage (2-3) and per patient using an inverted, light microscope (Olympus IMT-2), and analysed using Image-J for surface area (mm^2^). Clonogenicity should be evaluated once the organoid fragments re-close after splitting. In our human enteroid model, this process occured rapidly within 2 days after replating. Clonogenicity was therefore assessed at day 2 during three consecutive passages. During differentiation, enteroids were also classified according to their crypt development, which is based on the number of outward protruding crypts per enteroid (<5 crypt formations, 5-15 crypt formations and >15 crypt formations).

#### Quantitative real-time PCR (qRT-PCR)

The ReliaPrep miRNA Cell and Tissue Miniprep System (Promega) was used to isolate RNA from approximately 500 enteroids per treatment group. RNA from the mucosa was isolated using the RNeasy Mini Kit (Qiagen). Total RNA was reversed transcribed to cDNA using qScript cDNA SuperMix (Quantabio). Real time PCR was performed using the Lightcycler 480 with the Lightcycler 480 Sybr Green I Master mix (Roche Diagnostics). Primers (Sigma-Aldrich) are listed in Table S2. Raw data was analyzed using the software LinRegPCR to determine primer efficiency. All qRT-PCR results were normalized to a calibrator to correct for inter-run variations and to the geometric mean of the endogenous control genes B2M, S18 and RPS11, which were not affected by obesity or stimulants according to the method of Vandesompele.^51^

#### Whole-mount enteroid immunofluorescence staining

Enteroids were fixed with 4% paraformaldehyde at the end of differentiation and recovered from the matrix for whole-mount immunofluorescence staining.^52^ Antigen-retrieval was performed using 10 mM sodium citrate pH 6.0 for 30 min at 80°C followed by pre-incubation (2 h) with 10% donkey serum (Jackson ImmunoResearch) and 0.3% Triton X-100 (Sigma-Aldrich). Enteroids were then incubated overnight at 4°C with goat anti-chromogranin A (1:250; sc-1488, Santa Cruz), goat anti-ghrelin (1:250; sc-10368, Santa Cruz), rabbit anti-GLP-1 (1:100; ab108443, Abcam), rabbit anti-motilin (1:50; in-house developed antibody) or rabbit anti-Notch1 (1:100; D1E11, Cell signaling Technology). After washing, enteroids were stained during 2 h with a secondary antibody CY3 donkey anti-goat (1:800; 715-165-150, Jackson ImmunoResearch) or CY5 donkey anti-rabbit (1:800; 711-175-152, Jackson ImmunoResearch), depending upon the host of the primary antibody. Nuclei were stained with DAPI (2.5 μg/ml, ThermoFisher Scientific). For the negative control, the primary antibody was replaced with normal rabbit serum (Jackson ImmunoResearch). Enteroids were mounted back in a 96- CELLSTAR®-plate (Greiner) with Mowiol® 4-88 (Sigma-Aldrich) and visualized using the Operetta CLS High-Content Imaging system (Perkin Elmer). Counting was performed on at least 3-4 views from ± 10 enteroids per patient.

#### Ghrelin measurements

At the end of differentiation, enteroids were recovered from the matrix and incubated for 3 h with 0.75% peptone from pea (Fluka Analytical). The supernatant was collected and enteroids were lysed with lysis buffer (0.025 mM hyodeoxycholic acid, 1% v/v Igepal CA-630, 1 M Tris-HCl, 5 M NaCl, 1 tablet EDTA-free protease inhibitor cocktail (Thermofisher Scientific)). Both supernatants and enteroids were acidified with 10% 1 N HCl and 1% phenylmethylsulfonyl fluoride (10 mg/mL) was added. Samples were extracted on a Sep-Pak C18 cartridge (Waters Corporation) and vacuum-dried. Octanoyl ghrelin release was measured using an in-house radioimmunoassay as previously described.^53^ Ghrelin secretion in the supernatant was expressed as a fraction of the total hormone content per well and expressed relative to the secretion in the vehicle stimulated well which was run in parallel.

#### EdU proliferation assay

Proliferation was examined using The Click-iT® EdU Assay (Thermofisher Scientific) following the manufacturer’s instructions and visualized in the same manner as the whole-mount immunofluorescence staining of enteroids. The number of EdU+ cells were counted from 15 enteroids per patient and corrected for the surface area (mm^2^).

#### Permeability

Permeability of fluorescence markers into the lumen of enteroids was tested using fluorescein isothiocyanate (FITC)-dextran of 4 kDa and 40 kDa (TdB Labs AB). Enteroids were recovered from the matrix and incubated at 37°C with the respective fluorescent tracer at a final concentration of 0.5 mg/mL for 30 min. Marker solutions were removed and enteroid cultures were washed 3 times with PBS and fixed with CitiFluor™ (Thermofisher Scientific). Fluorescent labeling of the enteroid lumen was visualized using the widefield Olympus IX71 (VIB Bio-imaging Core Leuven). The number of enteroids (± 15) in which FITC-dextran (4 kDa) was present in the lumen was compared between control and SCFAs-treated wells per patient.

Permeability of fluorescence marker FITC-dextran of 4 kDa (TdB Labs AB) into the lumen of enteroids was also quantified. Enteroids were recovered from the matrix and incubated at 37°C with FITC dextran 4kDa at a final concentration of 10 mg/mL for 30 min. Marker solution was removed and enteroid cultures were washed 3 times with PBS. Enteroid pellets were then resuspended in a lysis buffer (0.025 mM hyodeoxycholic acid, 1% v/v Igepal CA-630, 1 M Tris-HCl, 5 M NaCl, 1 tablet EDTA-free protease inhibitor cocktail (Thermofisher Scientific)). The concentration of FITC dextran 4kDa in the enteroid lysate was then measured with the FLUOstar omega (BMG Labtech) using a standard curve.

### Quantification and statistical analysis

Results are presented as mean ± SEM. Log-transformation was performed on non-normally or non-homogeneously distributed data for further analyses.

The effect of obesity or stimulants on the morphology or the relative mRNA expression was analyzed using the SAS procedure Proc Mixed (SAS/Stat 15.2; SAS Institute, Cary, NC, USA) with ‘patient’ as random variable to examine both between- and within-group effects.

The effect of obesity on the relative mRNA expression of the transcription factors, cell proliferation assay, whole-mount immunofluorescent staining was analyzed using a non-paired 2-tailed Student’s t-test. The effect of stimulants on the relative mRNA expression of general gut epithelial cells (ALPI, MUC2 and CHGA), transcription factors, cell proliferation, whole-mount immunofluorescent staining, octanoyl ghrelin release and on permeability assays was analyzed using a paired 2-tailed Student’s t-test. The effect of obesity on cell proliferation, whole-mount immunofluorescent staining during expansion and differentiation was analyzed using non-paired, 2-tailed Student’s t-test. Differences in age and BMI between patients with obesity and normal weight individuals were analyzed using an non-paired, 2-tailed Student’s t test. Sex difference between populations was analyzed using the χ2 test in Statistica 13.3 (TIBCO Software, Inc.). Multiple testing was corrected using the (Holm-)Sidak method. Data was expressed as mean ± standard error of the mean (SEM). n is equal to the number of individuals. Significance was accepted at the 5% level (p-value = P).
