# Supplementary material for: SCFAs switch stem cell fate through HDAC inhibition to improve barrier integrity in 3D intestinal organoids from patients with obesity
Source: iScience. 2023 Nov 23;26(12):108517. doi: 10.1016/j.isci.2023.108517 (PMC10730380; doi:10.1016/j.isci.2023.108517)
Supplement: Document S1. Figures S1–S4 and Tables S1 and S2 [file mmc1.pdf]

iScience, Volume *26*

Supplemental information

SCFAs switch stem cell fate through HDAC inhibition to improve barrier integrity in 3D intestinal organoids from patients with obesity

Mona Farhadipour, Kaline Arnauts, Mathias Clarysse, Theo Thijs, Kathrin Liszt, Bart Van der Schueren, Laurens J. Ceulemans, Ellen Deleus, Matthias Lannoo, Marc Ferrante, and Inge Depoortere

SUPPLEMENTAL INFORMATION

Table S1: patient demographics from normal weight multi-organ donors and patients with obesity

PARAMETER	NORMAL WEIGHT (n=11)<br>(30% DCDIII, 70% DBD)	OBESE (n=11)	NORMAL WEIGHT vs. OBESE
Male/Female	9/3	4/7	NS*
Age (years)	52±4	39±3	P<0.05**
BMI (kg/m²)	23.8±0.4	41.8±1.2	P<0.001**

*Chi-square test ** Student's unpaired t-test

Table S2: list of primers used in RT-qPCR

GENE	ALIAS	FORWARD	REVERSE
Beta-2 Microglobulin	*B2M*	GCGCTACTCTCTCTTTCTGG	GCTGGATGACGTGAGTAAAC
Ribosomal Protein S18	*S18*	ACCAACATCGATGGGCGGCG	TGGTGATCACACGTTCCACCTCA
Ribosomal Protein S11	*RPS11*	CAGCCGACCATCTTTCAAAAC	TCTCGAAGCGGTTGTACTTG
Leucine-rich repeat-containing G-protein coupled receptor 5	*LGR5+*	CACCTCCTACCTAGACCTCAGT	CGCAAGACGTAACTCCTCCAG
HOP Homeobox	*HOPX*	GCCTCTTCCACCGCGCA	GACGGATCTGCACTCTGAGG
Olfactomedin 4	*OLFM4*	AGGTTCTGTGTCCCAGTTGT	CAAGCGTTCCACTCTGTCCA
SRY-Box Transcription Factor 9	*SOX9*	AGGAAGCTCGCGGACCAGTAC	GGTGGTCCTTCTTGTGCTGCAC
Hairy and Enhancer of Split-1	*HES1*	GGAAATGACAGTGAAGCACCTCC	GAAGCGGGTCACCTCGTTCATG
Atonal BHLH Transcription Factor 1	*ATOH1*	CGAGAGAGCATCCCGTCTAC	TCCGGGGAATGTAGCAAATA
Alkaline Phosphate, Intestinal	*ALPI*	TTCCTGGTGTCCCCACTTCG	TCCTCAGCTGGGATGACGC
Mucin 2	*MUC2*	CAGCACCGATTGCTGAGTTG	GCTGGTCATCTCAATGGCAG
Chromogranin A	*CHGA*	CCAAGGAGAGGGCACATCAG	TCTTCCACCGCCTCTTTCAG
Lysozyme	*LYZ*	ATCAGCCTAGCAAACTGGAT	CTCCACAACCTTGAACATAC
Ghrelin	*GHRL*	GGGCAGAGGATGAACTGGAA	CCTGGCTGTGCTGCTGGTA
Motilin	*MLN*	GGATGCAGGAAAAGGAACGG	CTGTCAGCAGCCCTTCCAG
Preproglucagon	*GCG*	CAAGGCAGCTGGCAACGT	TGGTGAATGTGCCCTGTGAA
Cholecystokinin	*CCK*	GAGGGTATCGCAGAGAACGG	GACGGCCAAAATCCATCCAG
Somatostatin	*SST*	ACCCAACCAGACGGAGAATGA	GCCGGGTTTGAGTTAGCAGA

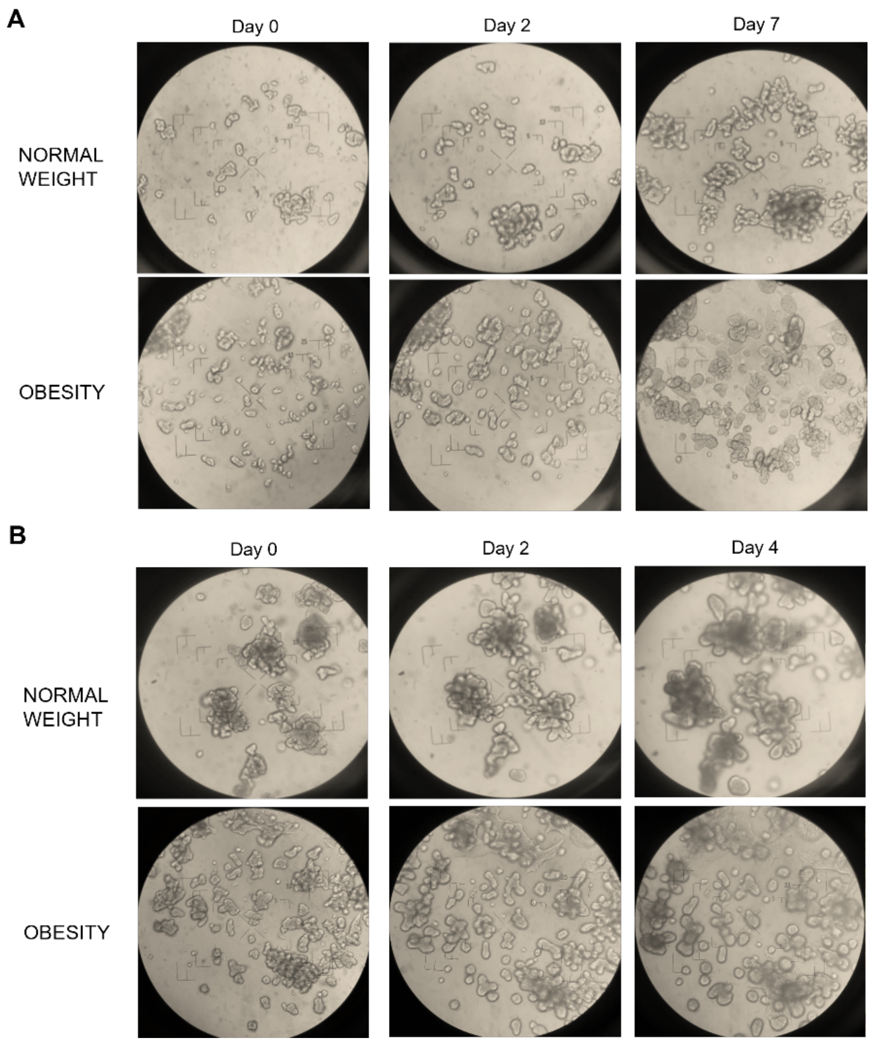

Fig. S1. Obesity affects the surface area of enteroids. Representative images of time-dependent changes in surface area of enteroids from both enteroid populations during (A) expansion and (B) differentiation. Enteroids from patients with obesity are significantly smaller than enteroids from normal weight individuals during both the expansion and differentiation phase.

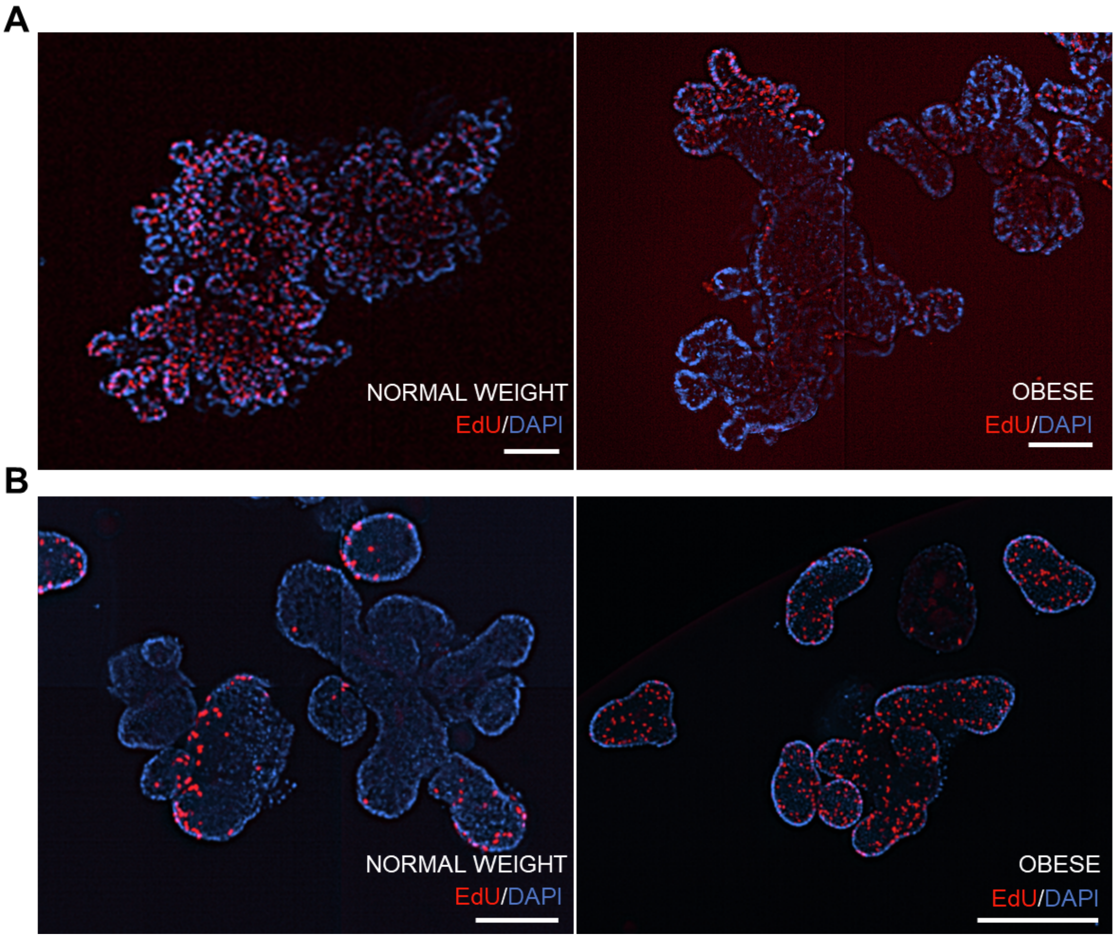

Fig. S2. Obesity affects proliferation of enteroids. Representative images of number of EdU^+ cells from both enteroid populations during (A) expansion and (B) differentiation. Proliferation in enteroids from patients with obesity is delayed and is higher than in enteroids from normal weight individuals in fully differentiated enteroids. Scale bar: 100 µm.

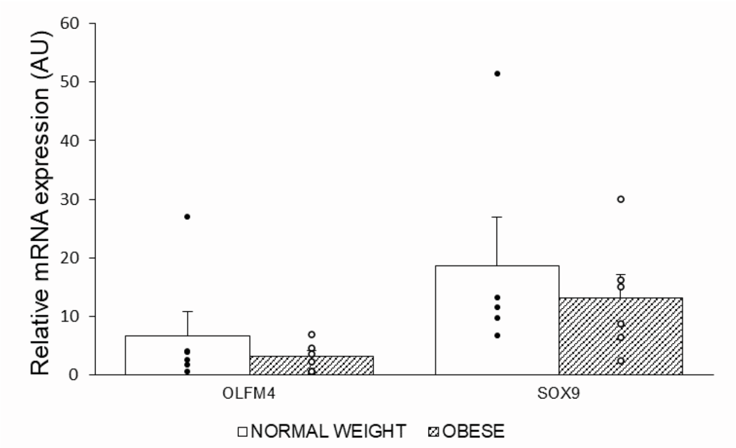

Fig. S3. Obesity does not affect *OLFM4* and *SOX9* mRNA expression. The relative mRNA expression ($2^{-\Delta\Delta Ct}$) of *OLFM4* and *SOX9* in both enteroids population ($n_{normalweight}$=5-6; n_{obese}=6) during the expansion phase. Statistical significance was determined using an unpaired 2-tailed Student's t-test. n is the number of individuals. Data are presented as mean ± SEM.

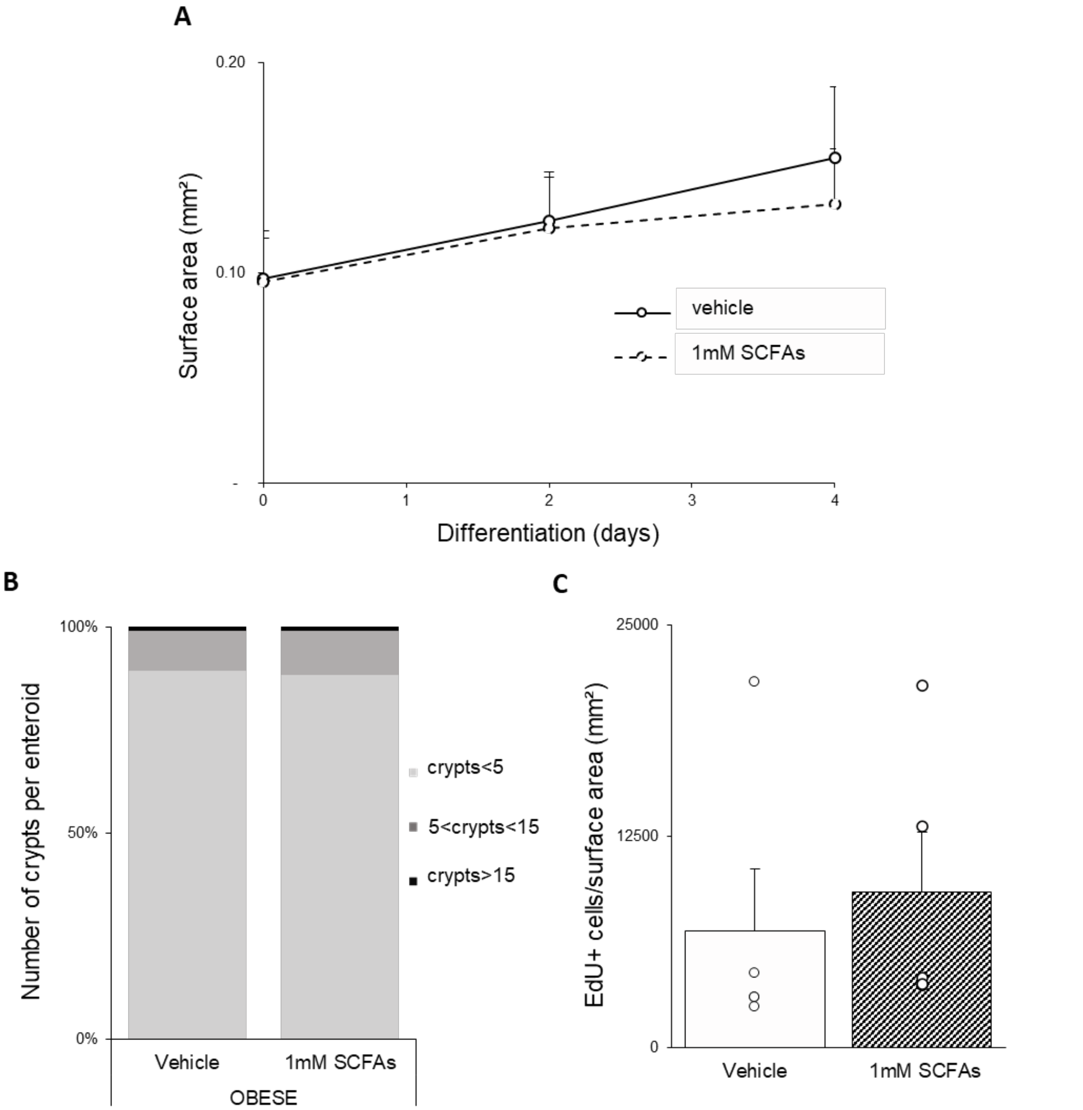

Fig. S4. SCFAs do not affect the morphology nor the proliferation of enteroids during the differentiation phase. (A) Time-dependent changes in surface area of enteroids from patients with obesity (n=3) after addition of 1 mM SCFAs during differentiation. (B) Effect of 1 mM SCFAs on the number of crypt formations per enteroid at day 4 of differentiation in the obese enteroid population (n=4). (C) Average number of EdU+ cells/surface area (mm^2) after 1 mM SCFAs treatment at the end of differentiation in enteroids from patients with obesity (n=5). Statistical significance was determined with a mixed model with patient as the random effect for surface area measurements, and a paired, 2-tailed Student's t-test for the EdU proliferation assay. n is the number of subjects. Data are presented as mean ± SEM.
